# Editorial: Cutting-edge technologies for the comprehensive analysis of neural circuits

**DOI:** 10.3389/fnana.2022.1101560

**Published:** 2022-12-05

**Authors:** Hirohide Iwasaki, Kea Joo Lee

**Affiliations:** ^1^Department of Anatomy, Gunma University Graduate School of Medicine, Maebashi, Japan; ^2^Neural Circuits Research Group, Korea Brain Research Institute, Daegu, South Korea; ^3^Department of Brain Science, Daegu Gyeongbuk Institute of Science and Technology, Daegu, South Korea

**Keywords:** neural circuit, light microscopy, electron microscopy, synapse, connectome

As the current topic, we targeted various innovative techniques and tools for a comprehensive analysis of neural circuits. The extensive worldwide “connectome” project attempts to elucidate the wiring diagram of neural circuits in detail (Swanson and Lichtman, 2016). Neural circuits are networks of multiple neurons that are connected *via* synapses. The detailed analysis of these networks requires both relatively extensive observation of the entire neuronal network at low resolution and high resolution microscopic observation of the synapses that form between individual neurons. Progress in computer sciences is also crucial for extracting information regarding neuronal connections from image datasets. As a result of many research projects, such as the Allen Brain Atlas project, a knowledge repository for the connectome is now available online. Methods for extracting valuable information from these datasets are also crucial for understanding neural circuits. Furthermore, along with realizing the neural circuits of the normal brain, such innovative technologies would be useful for elucidating the etiology and pathology of various psychiatric diseases, neurodegenerative diseases, and developmental disorders, as well. In this study, for each of the aforementioned topics, we present a wide range of related original papers and review articles.

Light microscopy is capable of acquiring multicolor images by harnessing light at various wavelengths, and has the feature of live imaging for obtaining data on living cells or individuals. In particular, recent years have unraveled the potential of viral vectors to label various specific neurons, leading to the greater comprehension of neural circuits. For this topic, we featured papers describing the usage of viral vectors that label neurons. Brown et al. introduced their dual-labeling method of combining fluoro-ruby and virus vectors to label long ascending propriospinal neurons (LAPNs) in the spinal cord. They used both adeno-associated viruses for retrograde labeling (retroAAV) and retro lentivirus (HiRet) for high efficiency, and compared the labeling efficiency and specificity of these viruses. Kler et al. described the improvement of Tracer with Restricted Anterograde Spread (TRAS), which they previously developed for the transsynaptic labeling of zebrafish neurons. In this paper, cytotoxicity was reduced by modifying the sequence of the vesicular stomatitis virus (VSV).

As living tissues can be analyzed by light microscopy, monitoring their neuronal activity is possible by using voltage indicators. Rhee et al. introduced a method to observe the spontaneous oscillations of the motor cortex by using brain slices expressing genetically encoded voltage indicators (GEVIs). They tried several GEVIs and compared which GEVIs were appropriate for monitoring spontaneous oscillations. They also demonstrated the different roles of both excitatory and inhibitory neurons in spontaneous oscillations.

Although light microscopy is a powerful tool for observing neurons at a relatively low resolution, some points are to be considered for the observations by light microscopy. One such problem is the chromatic aberration in observations when using multiple colors. In particular, chromatic aberration is problematic for the observation of large-volume samples such as brain tissues treated using tissue-clearing methods. Leiwe et al. introduced their methods to evaluate chromatic aberration in confocal laser microscopy with different objective lenses and developed a method for the *post-hoc* correction of chromatic aberration in large-scale volume images.

Although light microscopy can distinguish each neuron using several fluorescent probes with different fluorescence properties, there is a limitation in distinguishing each neuron by our eyes, even when using appropriate optical filters and dichroic mirrors. From this point of view, the approaches of DNA barcoding and transcriptomics are promising for precisely distinguishing each neuron than simply using light. Endo et al. introduced methods for spatial transcriptome analysis, such as *in situ* sequencing and *in situ* capturing technology, and also described lineage tracing methods using viruses. They also introduced innovative techniques such as expansion microscopy and transparency as examples of local circuit analysis in the cortex, and contrasted them with three-dimensional reconstruction techniques using electron microscopy.

Neural circuit analysis by electron microscopy has conventionally been based on the three-dimensional reconstruction of serial ultrathin sections using transmission electron microscopy. However, this method requires considerable time and technical skills, and the volume that can be reconstructed is limited to a relatively small size. Parajuli and Koike summarized various 3D reconstruction methods using scanning electron microscopy (SEM) methods, such as FIB-SEM, SBF-SEM, and ATUM-SEM. The morphological analysis of dendritic spines using these methods were described (Parajuli and Koike). Koga et al. introduced the application of SEM for neural structures. In particular, they introduced an osmium maceration method to observe the 3D ultrastructures of organelles in the nerve cells. They also introduced their approach for observing semi-thin sections of neural tissue using SEM. Schifferer et al. also introduced various methods for the observation of neural circuits, which were not limited to electron microscopies, but included various approaches, such as μCT and X-ray tomography. In their paper, the importance of precisely narrowing-down into the area to be reconstructed by EM to reduce time and labor was highlighted, along with combining the said approach for massive observation with low resolution. Correlative Light and Electron Microscopy (CLEM) is one such approach for integrating circuit analysis using light microscopy and electron microscopy. Iwasaki et al. introduced various probes for CLEM. They also mentioned the technique of merging images captured by electron microscopy and light microscopy, which is one of the critical problems for CLEM.

Computer-based approaches for information processing are essential for extracting information about structures from datasets reconstructed in three dimensions using electron microscopy. In particular, recent advances in machine learning, such as the development of algorithms for automatic segmentation, have increased the importance of computer-based approaches for the connectome. Park et al. developed a method for the automatic detection of synapses from EM images to elucidate the cerebellar connectome. Wang et al. introduced an approach for extracting information from the Allen Brain Atlas database. They focused on monoaminergic neuronal nuclei and analyzed the data using *in situ* hybridization. Kim et al. introduced iCANN, a method to experimentally verify neural computers by culturing neurons on microfabricated plates and artificially creating neural networks.

Based on these technological foundations, it becomes important to consider the kinds of neural circuits that need to be analyzed. The circuit of the cerebellum is relatively simple compared to other regions of the brain, such as the cerebral cortex, and the circuits of the cerebellar cortex have been studied precisely and are well-documented. Kang et al. summarize the latest findings on the output system of the cerebellum. The approaches introduced in this Research Topic are useful not only for understanding normal brain circuits but also for pathological purposes. Seo et al. used SBF-SEM to reconstruct cortical synapses in a 5xFAD mouse model and compared synaptic density between the medial pre-frontal cortex (mPFC) and the primary visual cortex (V1). They found reduced excitatory synaptic density only in the mPFC, but not in the V1 at the age of 6 months, which correlates with amyloid deposition levels. Intriguingly, synapses devoid of presynaptic mitochondria and the number of mitochondria per presynaptic bouton were significantly reduced in the mPFC of the 5xFAD mice. These results suggest new potential therapeutic targets for the prevention of early synaptic degeneration in AD.

In summary, our Research Topic covers most of the technologies related to connectomics. We hope that this Research Topic will help readers better understand various approaches to connectomics and that it will be helpful to researchers interested in neural circuits.

## Author contributions

HI and KL wrote the draft, edited, and finalized the manuscript. All authors contributed to the manuscript and approved the submitted version.
